# A case of left main coronary artery to pulmonary fistula associated with vasospastic angina

**DOI:** 10.1093/jscr/rjaf173

**Published:** 2025-04-05

**Authors:** Kentaro Shirakura, Shougo Takahashi, Kaname Shimizu, Jun Maruoka, Yuki Setogawa, Ryo Okubo, Hiroyuki Miyamoto, Ryohei Ushioda, Daisuke Takeyoshi, Shingo Kunioka, Masahiro Tsutsui, Hiroyuki Kamiya

**Affiliations:** Department of Cardiac Surgery, Asahikawa Medical University, Midorigaoka Higashi 2-1-1-1, Asahikawa 078-8510, Japan; Department of Cardiac Surgery, Asahikawa Medical University, Midorigaoka Higashi 2-1-1-1, Asahikawa 078-8510, Japan; Department of Cardiac Surgery, Asahikawa Medical University, Midorigaoka Higashi 2-1-1-1, Asahikawa 078-8510, Japan; Department of Cardiac Surgery, Asahikawa Medical University, Midorigaoka Higashi 2-1-1-1, Asahikawa 078-8510, Japan; Department of Cardiac Surgery, Asahikawa Medical University, Midorigaoka Higashi 2-1-1-1, Asahikawa 078-8510, Japan; Department of Cardiac Surgery, Asahikawa Medical University, Midorigaoka Higashi 2-1-1-1, Asahikawa 078-8510, Japan; Department of Cardiac Surgery, Asahikawa Medical University, Midorigaoka Higashi 2-1-1-1, Asahikawa 078-8510, Japan; Department of Cardiac Surgery, Asahikawa Medical University, Midorigaoka Higashi 2-1-1-1, Asahikawa 078-8510, Japan; Department of Cardiac Surgery, Asahikawa Medical University, Midorigaoka Higashi 2-1-1-1, Asahikawa 078-8510, Japan; Department of Cardiac Surgery, Asahikawa Medical University, Midorigaoka Higashi 2-1-1-1, Asahikawa 078-8510, Japan; Department of Cardiac Surgery, Asahikawa Medical University, Midorigaoka Higashi 2-1-1-1, Asahikawa 078-8510, Japan; Department of Cardiac Surgery, Asahikawa Medical University, Midorigaoka Higashi 2-1-1-1, Asahikawa 078-8510, Japan

**Keywords:** coronary artery fistula, vasospastic angina

## Abstract

Coronary artery fistulas (CAFs) are rare coronary anomalies. Among these, coronary artery-pulmonary artery fistulas can lead to myocardial ischemia through mechanisms such as coronary steal, stenosis, and vasospasm. We report a case of a 73-year-old male presenting with a coronary artery-pulmonary artery fistula and 75% stenosis of the right coronary artery. Despite negative findings for ischemia on myocardial scintigraphy and the absence of ST changes on a resting electrocardiogram (ECG), coronary steal syndrome was suspected following an exercise ECG that revealed diffuse ST depression. Surgical intervention to close the fistula was performed; however, the patient experienced intraoperative coronary spasms and ventricular fibrillation, necessitating the use of intra-aortic balloon pump and veno-arterial extracorporeal membrane oxygenation. The present case suggested that CAFs can be complicated by vasospasm, which may be overlooked at preoperative diagnostics. In patients with CAFs, careful evaluation regarding vasospastic angina should be done preoperatively.

## Introduction

Coronary artery fistulas (CAFs) are increasingly being detected due to the widespread use of coronary angiography. These fistulas are found in ~0.2% of adult patients undergoing coronary angiography for chest pain and other cardiac conditions [1, 2]. Although some reports indicate that CAF often drain into the right coronary artery (RCA), Gillebert et al. found that, in the majority of adult cases, the fistula originates from the proximal portion of the left anterior descending artery and opens into the main pulmonary artery trunk [2]. Chest pain associated with CAF is often atypical, and there are relatively few cases where myocardial ischemia can be demonstrated by exercise testing [3]. In cases of coronary pulmonary artery fistulas, the potential pathogenetic causes of myocardial ischemia include coronary steal, stenosis at the fistula site due to coronary atherosclerosis, and the involvement of coronary spasm [4–6].

We present a relatively rare case in which coronary steal caused by a CAF was complicated by coronary spasm.

## Case report

**Figure 1:**
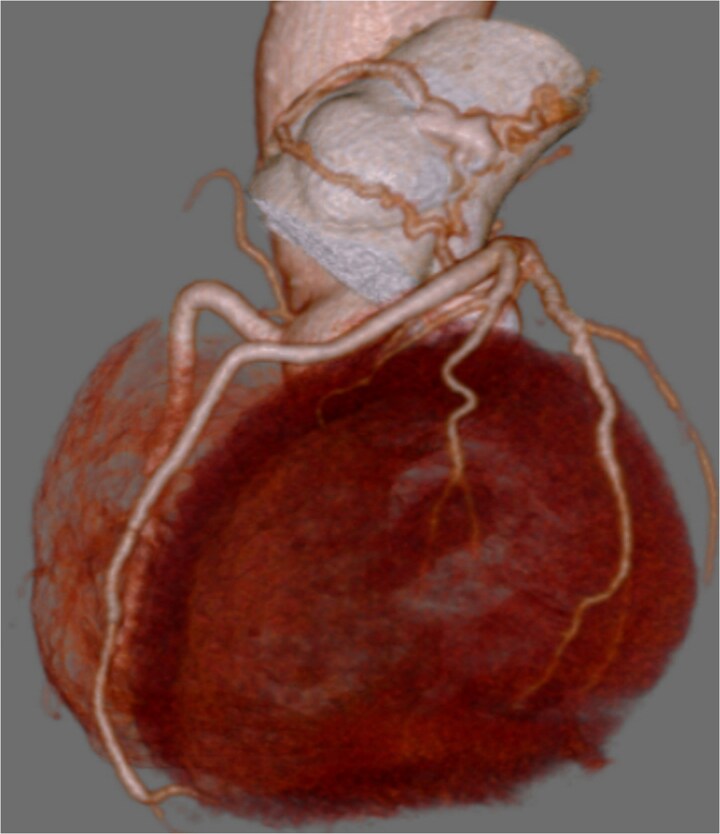
Preoperative coronary CT showing coronary artery-pulmonary artery fistula.

**Figure 2:**
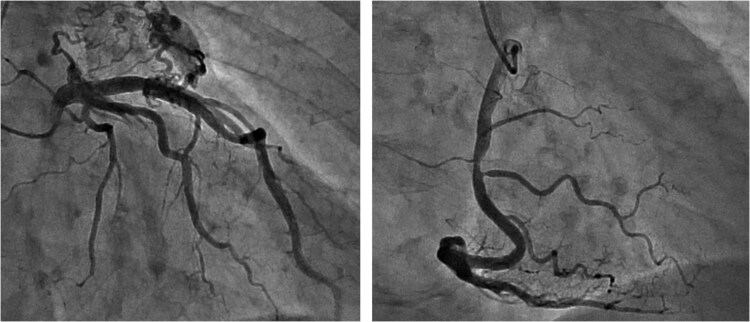
Preoperative CAG showing coronary artery-pulmonary artery fistula and 75% stenosis of the right coronary artery.

**Figure 3:**
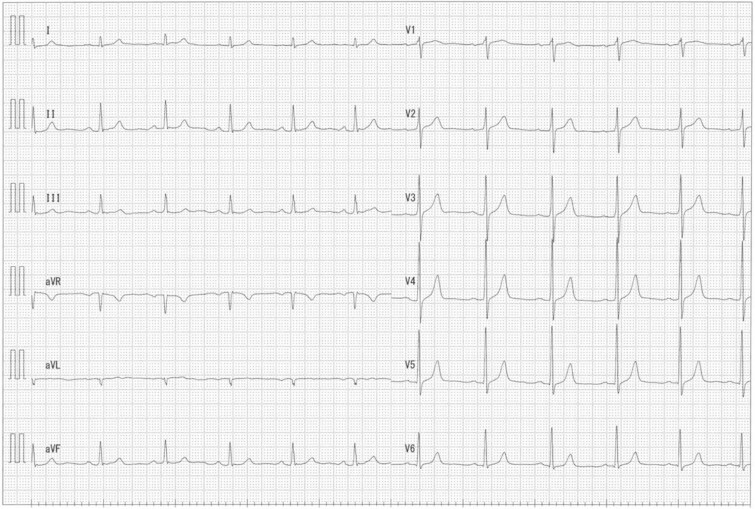
ECG at rest showing no ST changes.

**Figure 4:**
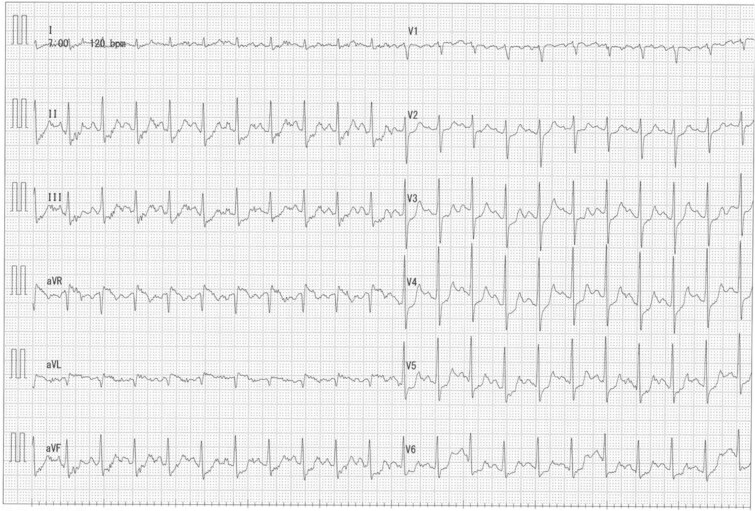
Exercise ECG showing diffuse ST depression.

A 73-year-old male presented to his primary hospital with complaints of chest pain. Examination revealed a coronary artery-pulmonary artery fistula and 75% stenosis of the RCA (Figs 1 and 2) with a resting full-cycle ratio (RFR) of 1.0. However, myocardial scintigraphy was negative for ischemia and electrocardiogram (CAG) at rest showed no ST changes (Fig. 3). An exercise resting electrocardiogram (ECG) showed diffuse ST depression, raising suspicion of ischemia due to coronary steal (Fig. 4). Consequently, the patient was referred to our department.

Transthoracic echocardiography revealed normal left ventricular function (62%). The flow from the fistula was not visualized in this study.

The surgery was performed through a median sternotomy with arterial cannulation into the ascending aorta and venous cannulation into the right atrium. After cardiac arrest with antegrade cardioplegia, a fistula was identified at the origin of the proximal left anterior descending artery (LAD) on the opposite side of the diagonal branch, which drained into the main pulmonary artery (PA). Entry point on the LAD was dissected and clipped. Subsequently, the orifice of the fistula into the PA was closed with 5–0 polypropylene via pulmonary arteriotomy.

**Figure 5:**
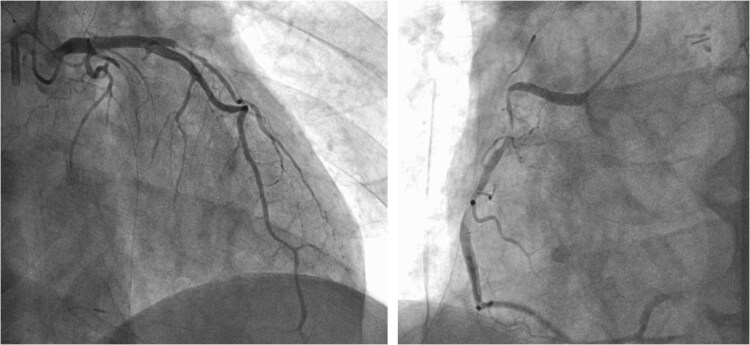
Intraoperative CAG revealing vasospasms along the entire length of the RCA and OM artery.

**Figure 6:**
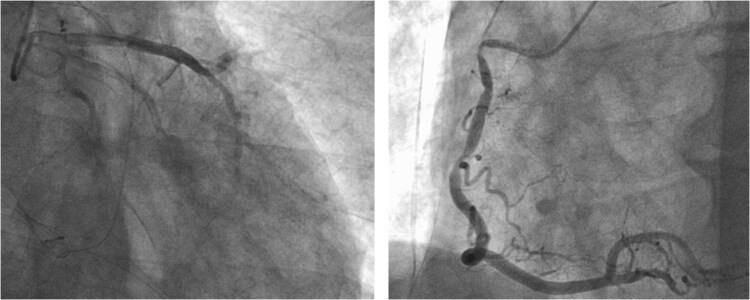
CAG after administration of nitroglycerin.

Following closure of the PA and cessation of cardiopulmonary bypass, ST-segment elevation was observed, accompanied by a deterioration in the patient's hemodynamics. Consequently, an intra-aortic balloon pump (IABP) was inserted via the right femoral artery and coronary angiography (CAG) revealed spasms along the entire length of the RCA and obtuse marginal artery (Fig. 5). These spasms improved generally after administration of nitroglycerin (Fig. 6). However, ventricular fibrillation was provoked, leading to unstable hemodynamics. Therefore, veno-arterial extracorporeal membrane oxygenation was initiated, and the surgery was concluded. Extracorporeal membrane oxygenation (ECMO) and IABP were withdrawn 4 days and 5 days after surgery, respectively. The patient's postoperative course was good, and he left the hospital on post-operative day 20.

## Discussion

A CAF is a rare anomaly of coronary anatomy, affecting ~0.002% of the general population. Most commonly congenital, CAFs can also be acquired through chest trauma, infection, or medical procedures such as surgery or cardiac catheterization [7]. Clinical presentation of CAFs ranges from asymptomatic to sudden cardiac death, with younger patients and those with smaller fistulas typically being asymptomatic. The most common symptoms include exertional dyspnea and angina, often due to the coronary steal phenomenon [3, 7–9]. The European Society of Cardiology and American Heart Association recommend that small, asymptomatic CAF be managed conservatively and monitored with echocardiograms every 3–5 years to assess for enlargement, degeneration, or symptomatic progression. Larger fistulas, due to their higher risk of complications, should be closed either percutaneously or surgically, even if asymptomatic. Small to moderate fistulas should only be closed if symptomatic or if complications arise, such as unexplained structural changes or dysfunction observed on echocardiography, a Qp/Qs ratio exceeding 1.5:1, or aneurysmal degeneration [10–12].

In this case, preoperative coronary angiography revealed 75% stenosis in the RCA and myocardial scintigraphy negative for ischemia. Additionally, exercise ECG showed no chest symptoms but significant ST depression, indicative of ischemia. The patient was diagnosed with coronary steal syndrome caused by a CAF and we decided to perform operation. In this case, atypical angina was not strongly suspected, as the patient's resting symptoms may have been masked by medications such as β-blocker, nicorandil, and isosorbide nitrate at the time of referral to our department. However, intraoperative findings suggest the possibility of a coronary spasm complication. Although the exact cause of myocardial ischemia remains unclear, some studies report an association between coronary pulmonary artery fistulas and vasospastic angina [13]. Therefore, it is crucial to consider the treatment plan carefully, taking into account the potential for rapid deterioration of circulatory dynamics.

## Conclusion

In addition to ischemia caused by coronary steal, complications of vasospastic angina, as seen in the present case, have also been reported in CAF. In patients with CAFs, careful evaluation regarding vasospastic angina should be done preoperatively.
